# *In vitro* Spermatogenesis – Optimal Culture Conditions for Testicular Cell Survival, Germ Cell Differentiation, and Steroidogenesis in Rats

**DOI:** 10.3389/fendo.2014.00021

**Published:** 2014-02-26

**Authors:** Ahmed Reda, Mi Hou, Luise Landreh, Kristín Rós Kjartansdóttir, Konstantin Svechnikov, Olle Söder, Jan-Bernd Stukenborg

**Affiliations:** ^1^Pediatric Endocrinology Unit Q2:08, Department of Women’s and Children’s Health, Karolinska Institutet and University Hospital, Stockholm, Sweden

**Keywords:** testis, spermatogenesis, cell culture, culture medium, Leydig cells, testosterone, stem cell niche

## Abstract

Although three-dimensional testicular cell cultures have been demonstrated to mimic the organization of the testis *in vivo* and support spermatogenesis, the optimal culture conditions and requirements remain unknown. Therefore, utilizing an established three-dimensional cell culture system that promotes differentiation of pre-meiotic murine male germ cells as far as elongated spermatids, the present study was designed to test the influence of different culture media on germ cell differentiation, Leydig cell functionality, and overall cell survival. Single-cell suspensions prepared from 7-day-old rat testes and containing all the different types of testicular cells were cultured for as long as 31 days, with or without stimulation by gonadotropins. Leydig cell functionality was assessed on the basis of testosterone production and the expression of steroidogenic genes. Gonadotropins promoted overall cell survival regardless of the culture medium employed. Of the various media examined, the most pronounced expression of *Star* and *Tspo*, genes related to steroidogenesis, as well as the greatest production of testosterone was attained with Dulbecco’s modified eagle medium + glutamine. Although direct promotion of germ cell maturation by the cell culture medium could not be observed, morphological evaluation in combination with immunohistochemical staining revealed unfavorable organization of tubules formed *de novo* in the three-dimensional culture, allowing differentiation to the stage of pachytene spermatocytes. Further differentiation could not be observed, probably due to migration of germ cells out of the cell colonies and the consequent lack of support from Sertoli cells. In conclusion, the observations reported here show that in three-dimensional cultures, containing all types of rat testicular cells, the nature of the medium *per se* exerts a direct influence on the functionality of the rat Leydig cells, but not on germ cell differentiation, due to the lack of proper organization of the Sertoli cells.

## Introduction

Male infertility, a common disorder, is associated with a wide spectrum of spermatogenic failures, an increasing number of which are iatrogenic effects of clinical treatment (1). Treatment of children with cancer, including radiotherapy and high-dose chemotherapy, can severely damage the immature gonads and lead to infertility later in life (2). Since long-term survival of pre-pubertal patients with cancer has risen by as much as 80% during recent decades (3–5), more infertile patients can be expected in the future.

One approach to developing ways to rescue the fertility of these and other infertile patients is *in vitro* characterization of spermatogenesis, utilizing systems that mimic the natural situation as closely as possible and provide functional testicular cells for analyses (6).

In three-dimensional cultures of murine Sertoli, Leydig, peritubular, and germ cells stimulated with gonadotropins, pre-meiotic germ cells differentiate into postmeiotic spermatids, but with very low efficiency (6–8). Clearly, the optimal conditions for such cultures remain to be elucidated. In three-dimensional cultures containing all murine testicular cells, testosterone production by the Leydig cells was enhanced in response to stimulation by hCG for as long as 16 days (6). It remains to be determined whether similar Leydig cell function can be achieved with testicular cells from other species, including humans, under the same conditions.

To date, only traditional media, i.e., Dulbecco’s modified eagle medium (DMEM) medium, F12, and minimal essential medium (MEM), have been employed for culturing testicular cells (9, 10). Even though it is well established that gonadotropins play a pivotal role in spermatogenesis and that functioning Leydig and other somatic cells are important for the spermatogenic process (7, 11–13), optimal culture conditions for the different types of testicular cells, and for appropriate paracrine interactions between these cells have not yet been determined.

Accordingly, in the present investigation we attempted to create an optimal culture system, of endocrine and paracrine stimulation focusing on the nutritional requirements for appropriate development of three-dimensional cultures of rat testicular cells. More specifically, we assessed germ cell differentiation, tubule formation, Leydig cell functionality, and cell survival in cultures hosting all of the testicular cells, i.e., Sertoli, Leydig, peritubular, and germ cells.

## Materials and Methods

### Animals

Male Sprague-Dawley rats at 7 days of *post-partum* (d*pp*) age were purchased from Charles River (Sulzfeld, Germany) and transported to Karolinska Institutet (Stockholm, Sweden) together with their mothers. Each experiment involved testicular material from several different litters of these pups. Their use and handling was pre-approved by the ethics committee for experimental laboratory animals at Karolinska Institutet (N489/11).

### Tissue and cell preparation

The rat pups were sacrificed by decapitation and their testes immediately placed in DMEM containing glutamine (P/N 41966, Gibco, CA, USA) and supplemented with 1% penicillin/streptomycin (pen/strep; P/N 15070, Gibco). Single-cell suspensions were obtained by the three-step enzymatic digestion described previously (14). In brief, the first digestion was performed with Collagenase/Dispase (P/N 269638, Roche, Switzerland, Basel; final concentration: 0.04/0.32 U/ml) in DMEM for 10 min at 32°C with shaking at 120 rpm, followed by centrifugation at 100 × *g* for 2 min. The resulting supernatant was centrifuged again at 200 × *g* for 8 min and the cell pellet thus obtained re-suspended in DMEM and stored on ice.

The second digestion was accomplished with Collagenase/Dispase + DNAse (P/N 104159, Roche; final concentrations: 0.04/0.32 and 48 U/ml, respectively) in DMEM for 15 min at 32°C with shaking at 120 rpm, followed by centrifugation at 100 × *g* for 2 min. Centrifugation of the supernatant for 8 min at 200 × *g* provided the second cell pellet, which was also re-suspended in DMEM and stored on ice.

The third digestion of remaining tissue involved Collagenase/Dispase + DNAse + Collagenase IV (P/N C-1889, Sigma-Aldrich, St. Louis, USA; final concentrations: 0.04/0.32, 48 and 50 U/ml, respectively) in DMEM for 20 min at 32°C with shaking at 120 rpm, followed by collection and re-suspension of the third cell pellet in the same manner as above. All three cell suspensions were pooled, centrifuged at 200 × *g* for 8 min, re-suspended in 1 ml DMEM, counted in a Bürker chamber, and examined for viability by trypan blue staining (P/N 15250061, Gibco; 1:20 dilution).

### Cell cultures

As stated in Table 1, the different media tested here were DMEM + glutamine or without glutamine (DMEM − glutamine; P/N 21969, Gibco), DMEM + Glutamax (P/N 31966, Gibco), DMEM/F12 (P/N 21331, Gibco), F12 (P/N 21765, Gibco), and MEM (P/N 21430, Gibco). Pre-pubertal rat testicular cells were cultured in an agarose-medium matrix in accordance with previous reports (7). In brief, this matrix was prepared by mixing autoclaved 0.7% SeaKem^®^ LE agarose (P/N 50004, Lonza, Basel, Switzerland) or 0.7% LMP agarose (P/N 15517022, Invitrogen, CA, USA) with the relevant culture medium (supplemented with 1% pen/strep) at a ratio of 1:1 to give a final agarose concentration of 0.35% agarose.

**Table 1:** **Schematic illustration of the experimental conditions employed to characterize the effects of the culture medium and gonadotropins on three-dimensional cultures of testicular cells**.

Medium	Supplement			
	AA (%)	NEAA (%)	rFSH (IU/l)	hCG (IU/l)
DMEM (high glucose, +pyruvate, +l-glutamine; P/N 41966, Gibco)	–	–	5.0	5.0
	–	–	–	–
DMEM (high glucose, +pyruvate,−l-glutamine; P/N 21969, Gibco)	–	–	5.0	5.0
	–	–	–	–
DMEM (high glucose, +pyruvate, +Glutamax; P/N 31966, Gibco)	–	–	5.0	5.0
	–	–	–	–
F12 (+l-glutamine; P/N 21765, Gibco)	–	–	5.0	5.0
	–	–
	4.0	–	5.0	5.0
	–	–
	–	4.0	5.0	5.0
	–	–
	4.0	4.0	5.0	5.0
	–	–
DMEM/F12 (without l-glutamine; P/N 21331, Gibco)	–	–	5.0	5.0
	–	–	–	–
MEM (without l-glutamine; P/N 21430, Gibco)	–	–	5.0	5.0
	–	–	–	–

*AA, amino acids; NEAA, non-essential amino acids; rFSH, recombinant follicle-stimulating hormone; hCG, human chorionic gonadotropin; IU/l, international units per liter; M, molar mass (kg/mol); DMEM, Dulbecco modified Eagle’s medium; MEM, minimal essential medium; − = none*.

These cultures were exposed to recombinant follicle-stimulating hormone [rFSH; P/N Gonal F 75 IE, Merck, Frankfurt, Germany; final concentration: 5I U/l (international units per liter)] and human chorionic gonadotropin (hCG; P/N Pregnyl 5000 IE, Merck Sharpe and Dohme, NJ, USA; final concentration: 5I U/l) as also described in Table 1. The influence of amino acids on testosterone production were evaluated by adding essential amino acids (AA; P/N 11130-036, Gibco) or non-essential amino acids (NEAA; P/N 11140-035, Gibco) separately to F12 medium at a final concentration of 4%, similar to their concentrations in DMEM.

The single-cell suspensions (1.0 × 10^6^ cells/ml) were inoculated into the agarose-medium matrix before it solidified. To study cell migration, individual cell colonies, containing 50–100 cells each, were aspirated into a 22S-gage Hamilton syringe (P/N 80665/00, Hamilton Bonaduz AG, Bonaduz, Switzerland), placed separately onto six-well culture dishes (Gibco) containing DMEM (a high concentration of glucose + pyruvate, + l-glutamine; P/N 41966, Gibco) and cultured for as long as 5 days without changing the medium. All cell cultures were maintained at 35°C under 5% CO_2_ and performed in triplicates.

### Immunohistochemical, immunofluorescent, and morphological analyses

Testicular tissue and cell cultures were fixed in 4% paraformaldehyde (PFA; P/N15812-7, Sigma-Aldrich) overnight at 4°C, followed by serial dehydration in 30, 50, and 70% aqueous ethanol (24 h at each concentration) at room temperature (RT). Thereafter, the samples were placed for 6 h each in 80, 96, and 99.6% ethanol at RT, followed by soaking in 100% butyl acetate for 6 h at RT (P/N 45860, Sigma-Aldrich). Subsequently, these samples were embedded in paraffin (Paraplast X-TRA^®^; P/N P3808, Sigma-Aldrich) at 61°C overnight in standard fashion; cut into 5–20 μm slices using a Biocut sectioning machine (Reichert-Jung, NY, USA) and then placed on microscope slides (P/N10143352, Superfrost Plus, Thermo Scientific, MA, USA).

For immunohistochemical (IHC) and immunofluorescent (IF) staining, these samples were next de-paraffinized with xylene (P/N 02080, HistoLab, Gothenburg, Sweden) for 10 min and then serially rehydrated with 99.6, 96, and 70% aqueous ethanol, each step being performed twice for 5 min. After washing twice with phosphate-buffered saline (PBS, pH 7.4; P/N 14190-094, Gibco), antigen retrieval was achieved either by incubation with 0.1% sodium citrate (P/N S4641, Sigma-Aldrich) and 0.1% Triton X-100 (P/N 11869, Merck) in PBS for 8 min at RT or by heating for 15 min in 0.1 M sodium citrate buffer (P/N S4641, Sigma-Aldrich; pH 6) in a microwave oven at 600 W. Blocking was performed for 20 min at RT with 5% goat serum (P/N S-1000, VECTOR, CA, USA) or 5% donkey serum (P/N 017-000-121, Jackson ImmunoResearch, West Grove, PA, USA), depending on the secondary antibody employed, in 0.1% BSA (Bovine serum albumin; P/N A4503, Sigma-Aldrich) in PBS.

Rabbit polyclonal anti-Ddx4 antibody (also known as Vasa; P/N ab13840, Abcam, Cambridge, UK, 1:200 dilution, final concentration 5 μg/ml) in PBS containing 0.1% BSA was used for IHC staining, with non-specific rabbit IgGs (P/N ab27478, Abcam, final concentration 5 μg/ml and P/N sc-2027, Santa Cruz, CA, USA, final concentration 5 μg/ml) as negative controls. Polyclonal rabbit anti-Ap-2gamma (Ap-2γ; P/N sc-8977, Santa Cruz, 1:100 dilution, final concentration 2 μg/ml in PBS containing 0.1% BSA) was utilized for immunofluorescence staining, again with rabbit IgGs (P/N sc-2027, Santa Cruz, final concentration 2 μg/ml) as negative controls.

After incubation with the primary antibodies or control IgGs at 4°C overnight and three subsequent washes at RT with PBS, samples were stained immunohistochemically with biotinylated goat anti-rabbit IgG secondary antibodies (P/N ab64256, Abcam, final concentration 5 μg/ml) at RT for 2 h; then, washed three times with PBS, incubated with ABC reagents (P/N PK-6100, VECTOR); and developed with DAB (Diaminobenzidine; SK-4100, VECTOR). These slides were counterstained with hematoxylin (Mayer’s Hemalaun solution; P/N 1092491000, Merck), serially dehydrated with increasing aqueous ethanol solutions and then 100% xylene, and mounted with Entellan^®^ new (P/N 1079610100, Merck). For IF staining, samples were incubated with a Cy^3^-conjugated donkey anti-rabbit IgG secondary antibody (P/N 711-166-152, Jackson ImmunoResearch, West Grove, PA, USA, 1:600 dilution, final concentration 2.5 μg/ml) at RT for 1 h and the slides then counterstained and mounted with VECTASHIELD mounting medium containing DAPI (P/N H-1500, VECTOR).

For IF double-staining, paraffin-embedded samples on slides were first de-paraffinized with xylene for 10 min and then gradually rehydrated with 99.6, 96, and 70% ethanol, each step being performed twice for 5 min as described above. Staining was achieved employing the protocol described by van den Driesche and colleagues (15). In brief, for antigen retrieval, slides were treated with 0.01 M sodium citrate buffer, pH 6.0, containing 0.05% Tween 20 (P/N 8.17072.1000, Merck) at 96°C for 20 min in a water bath and thereafter blocked with 3% H_2_O_2_ (P/N 1.07209.0250, Merck) dissolved in methanol (P/N 1.06009.2511, Merck) for 30 min at RT. After two 5-min washes in Tris-buffered saline (TBS; P/N sc-24951, Santa Cruz), the sections were again blocked using 20% chicken serum (P/N C5405, Sigma-Aldrich) in TBS containing 5% BSA (P/N 001-000-161 Jackson ImmunoResearch) (TBS/NChS/BSA).

Subsequently, rabbit polyclonal primary antibodies against Ddx4 (P/N ab13840, Abcam, 1:200 dilution, final concentration 5 μg/ml), rabbit monoclonal primary antibodies against vimentin (P/N ab92547, Abcam, 1:200 dilution, final concentration 5 μg/ml), rabbit polyclonal primary antibodies against 3βHSD (P/N sc-28206, Santa Cruz, 1:200 dilution, final concentration 1 μg/ml) or rabbit IgGs (negative control) (P/N ab27478, Abcam, final concentration 5 μg/ml), all diluted in TBS/NChS/BSA, were incubated with the samples at 4°C overnight. The slides were then washed in TBS three times for 5 min each, followed by incubation with peroxidase-conjugated chicken secondary anti-rabbit antibody (P/N sc-2963, Santa Cruz, 1:200 dilution, final concentration 2 μg/ml) in TBS/NChS/BSA for 30 min at RT. After again washing in TBS three times for 5 min each, the Tyramide Fl kit (Perkin-Elmer-TSA plus Fluorescein System; P/N NEL741001KT, Perkin Elmer Life Sciences, Boston, USA) was employed in accordance with the manufacturer’s instructions. After washing once more with TBS, the sections were blocked again with 3% H_2_O_2_ in TBS–Tween for 30 min at RT, followed by blocking in TBS/NChS/BSA for 30 min at RT.

Thereafter, the sections were incubated with polyclonal rabbit primary anti-Ki67 antibodies (P/N ab27478, Abcam, dilution 1:200, final concentration 5 μg/ml) or rabbit IgGs (negative control) (P/N ab27478, Abcam, final concentration 5 μg/ml), both diluted in TBS/NChS/BSA, at 4°C overnight. Following washing with TBS, the samples were then incubated with peroxidase-conjugated chicken secondary anti-rabbit antibody (P/N sc-2963, Santa Cruz, 1:200 dilution, final concentration 2 μg/ml) dissolved in TBS/NChS/BSA for 30 min at RT. After again washing with TBS, the Tyr–Cy5 system (Perkin-Elmer-TSA plus Cyanine3 System; P/N NEL744001KT, Perkin Elmer Life Sciences) was applied in accordance with the manufacturer’s protocol and the slides subsequently mounted in VECTASHIELD mounting medium containing DAPI (P/N H-1500, VECTOR).

The different types of male germ cells were identified on the basis of morphological characteristics described previously: spermatogonia: round to oval nucleus with densely stained chromatin; leptotene spermatocytes: round with chromatin “speckled” nucleus; early pachytene spermatocytes: slightly larger nucleus containing chromatin cords throughout (16).

All stained sections were examined under an Eclipse E800 microscope (Nikon, Japan, Tokyo) and photographed with a 12.5 million-pixel cooled digital color camera system (Olympus DP70, Tokyo, Japan).

### Staining of apoptotic cells

To evaluate the influence of the various media on the viability of testicular cells *in vitro,* apoptosis was assessed using the TUNEL (Terminal deoxynucleotidyl transferase dUTP nick end-labeling) assay kit (DeadEnd™ Colorimetric Tunel System, P/N G7130, Promega, WI, USA) in accordance with the protocol provided. In brief, cell cultures were fixed in 4% PFA (P/N 8187081000, Merck) overnight at 4°C, followed by serial dehydration in 30, 50, and 70% aqueous ethanol for 24 h each. The samples were then transferred into 80, 96, and 99.6% ethanol for 6 h each at RT, followed by soaking in 100% butyl acetate for 6 h at RT (P/N 45860, Sigma-Aldrich) and, thereafter, routine embedding in paraffin (Paraplast X-TRA^®^; P/N P3808, Sigma-Aldrich) at 61°C overnight. After being cut into 5–20 μm slices using a Biocut sectioning machine (Reichert-Jung, NY, USA) and placed on microscope slides (P/N 10143352, Superfrost Plus, Thermo Scientific, MA, USA), the paraffin-embedded samples were de-paraffinized with xylene for 10 min; serially rehydrated with 99.6, 96, and 70% aqueous ethanol, with each step being performed twice for 5 min, and then washed twice with PBS.

Thereafter, the samples were treated with proteinase K (20 μg/ml in PBS) for 20 min at RT; washed again with PBS; and then incubated with biotinylated nucleotide mix + rTDT enzyme + buffer at 37°C for 1 h (adding only biotinylated nucleotide mix to the negative control). After terminating the reaction with stopping buffer (provided with the kit) and washing in PBS, endogenous peroxidase was blocked using 0.3% hydrogen peroxide (also supplied with the kit) in PBS for 15 min at RT. The samples were then incubated with streptavidin–HRP (from the kit) for 30 min at RT, stained with DAB (from the kit); counterstained with hematoxylin (Mayer’s Hemalaun solution; P/N 1092491000, Merck); dehydrated with increasing concentrations of aqueous ethanol and then 100% xylene; and mounted with Entellan^®^ new (P/N 1079610100, Merck). By examining at least 500 cells in each sample under an ECLIPSE E800 microscope (Nikon), the percentage of TUNEL-positive (i.e., apoptotic) cells was finally determined. The apoptotic frequency is expressed relative to the corresponding frequency on the first day of culturing, in order to minimize the effect of possible differences in culturing techniques.

### Testosterone assay

Testosterone production following 0, 1, 7, and 14 days of culture was employed as a measure of the influence of various media on the functionality of Leydig cells. First, testosterone was extracted by adding 0.5 ml ethyl acetate (P/N 1096232500, Merck) to the culture samples, each in a 1.5 ml Eppendorf tube, followed by vigorous automatic shaking for 15 min. After centrifugation for 2 min at 16000 × *g*, the resulting supernatant was re-subjected to the same procedure. The two ethyl acetate extracts were combined and evaporated overnight; the pellet obtained dissolved in PBS and the COAT-A-COUNT^®^ kit (P/N TKTT2, Siemens, Germany, Munich) used to quantify testosterone in accordance with the manufacturer’s protocol.

### RNA extraction and cDNA synthesis

Employing samples collected at the time-points designated and stored thereafter at −80°C, RNA was extracted as described previously (17). In brief, each sample was lysed with TRIzol^®^ reagent (P/N 15596018, Invitrogen) and disrupted for 30 s in an ULTRA-TURRAX T25 homogenizer (JANKE and KUNKEL, Staufen, Germany). Following addition of chloroform (P/N 1024452500, Merck) and centrifugation at 16000 × *g* for 10 min at 4°C, a half volume of ethanol 100% was added to the aqueous upper phase containing the RNA and the sample then applied to the spin column of the RNeasy Mini Kit (P/N 74104, Qiagen, Venlo, Netherlands) in accordance with the manufacturer’s protocol. The RNA thus isolated was treated with DNase 1 Amplification Grade (P/N AMPD1, Sigma-Aldrich) to eliminate contamination by DNA and thereafter 0.6 μg RNA from each sample were used to synthesize 20 μl cDNA with the IScript™ cDNA synthesis kit (P/N 170-8891, Bio-Rad, CA, USA) as instructed by the manufacturer.

### Analysis of gene expression

The influence of the various culture media on steroidogenesis and male germ cell differentiation was examined by analyzing relative gene expression by quantitative PCR (qPCR).

To assess steroidogenic gene expression, the iQ SYBER^®^ Green Super mix (P/N 170-8882, Bio-Rad) was employed as instructed and qPCR performed with the iCycler iQ multicolor RT-PCR detection system (Bio-Rad). The qPCR program was initiated with denaturation (3 min at 96°C); followed by 40 cycles of denaturation (96°C for 10 s) and annealing/elongation (60°C for 45 s). Two genes expressed specifically by rat Leydig cells – i.e., those encoding steroidogenic acute regulatory protein (*Star*) and peripheral benzodiazepine receptor or translocator protein (*Tspo*) – were examined, with beta actin (*Actb*) as the endogenous control. The qPCR efficiencies for *Star*, *Tspo*, and *Actb* were 87.6, 85.8, and 94.2%, respectively. All primer sequences and product sizes are documented in Table 2. The mean gene expression for the triplicates run in each medium was calculated by the ddCt procedure and then normalized to the mean level of *Actb* mRNA (dCt). Freshly isolated cells inoculated into agarose without gonadotropins were snap frozen immediately and the gene expression in each sample presented relative to the corresponding expression in these day-0 cells [fold-change (2^−ddCT^)].

**Table 2:** **The primers and conditions used for qPCR**.

Gene	Primer sequence 5′–3′	Amplicon size (bp)	Conditions
*Star*	Fw: CTGCTAGACCAGCCCATGGAC	90	40 cy
	Rev: TGATTTCCTTGACATTTGGGT		60°C
*Tspo*	Fw: GCTATGGTTCCCTTGGGTCT	195	40 cy
	Rev: GGCCAGGTAAGGATACAGCA		60°C
*Actb*	Fw: TGAAGATCAAGATCATTGCTC	120	40 cy
	Rev: ACTCATCGTACTCCTGCTTGC		60°C

*Bp, basepair; cy, cycles*.

In the case of male germ cell differentiation *in vitro*, TaqMan^®^ probes and TaqMan^®^ Gene Expression Master Mix (P/N 4369510, Applied Biosystems, Life technologies, CA, USA) were employed using the protocol suggested. In brief, utilizing the iCycler iQ multicolor RT-PCR detection system (Bio-Rad), the qPCR program started with 2 min at 50°C; then 10 min at 95°C; followed by 45 cycles of two steps; 15 s at 95°C and 1 min at 60°C. Six genes, expressed specifically in connection with germ cell differentiation were investigated, i.e., *Kit, Zbtb16* (zinc finger- and BTB-domain containing 16), *Dazl* (deleted in azoospermia-like), *Boll* [Boule-like (*Drosophila*)], *Crem* (cAMP responsive element modulator), and *Prm1* (protamine 1). The TaqMan^®^ probes utilized and assay numbers are listed in Table 3. The mean gene expression for the three triplicates run in each medium was calculated by the ddCt procedure and normalized to the corresponding mean level of *Actb* mRNA (dCt). The gene expression in each sample is presented relative to the corresponding expression in DMEM + glutamine [fold-change (2^−ddCT^)].

**Table 3:** **The assay and conditions used for qPCR**.

Gene	TaqMan^®^ assay number	Conditions
*Kit*	Rn00573942_m1	45 cy
		60°C
*Zbtb16*	Rn01418644_m1	45 cy
		60°C
*Dazl*	Rn01757162_m1	45 cy
		60°C
*Boll*	Rn01441407_m1	45 cy
		60°C
*Crem*	Rn01538528_m1	45 cy
		60°C
*Prm1*	Rn02345725_g1	45 cy
		60°C
*Actb*	Rn00667869_m1	45 cy
		60°C

*Cy, cycles*.

### Statistical analyses

Gene expression, apoptotic frequency, and testosterone production were calculated as the means ± standard deviations (SD) for the triplicates run under each condition. Student’s *t*-test, One-way ANOVA and One-way RM ANOVA were applied to compare the differences between experimental conditions (SigmaPlot 11.0; Systat Software Inc., CA, USA). Following the Shapiro–Wilk test for normality, pairwise multiple comparisons were performed with the “Holm–Sidak” procedure as stated in the Figure legends (SigmaPlot 11.0; Systat Software Inc.). A difference was considered to be statistically significant if the *p* value was ≤0.05.

## Results

### Influence of the various culture media and gonadotropins on the capacity of Leydig cells in a three-dimensional culture to produce androgens

**Figure 1:**
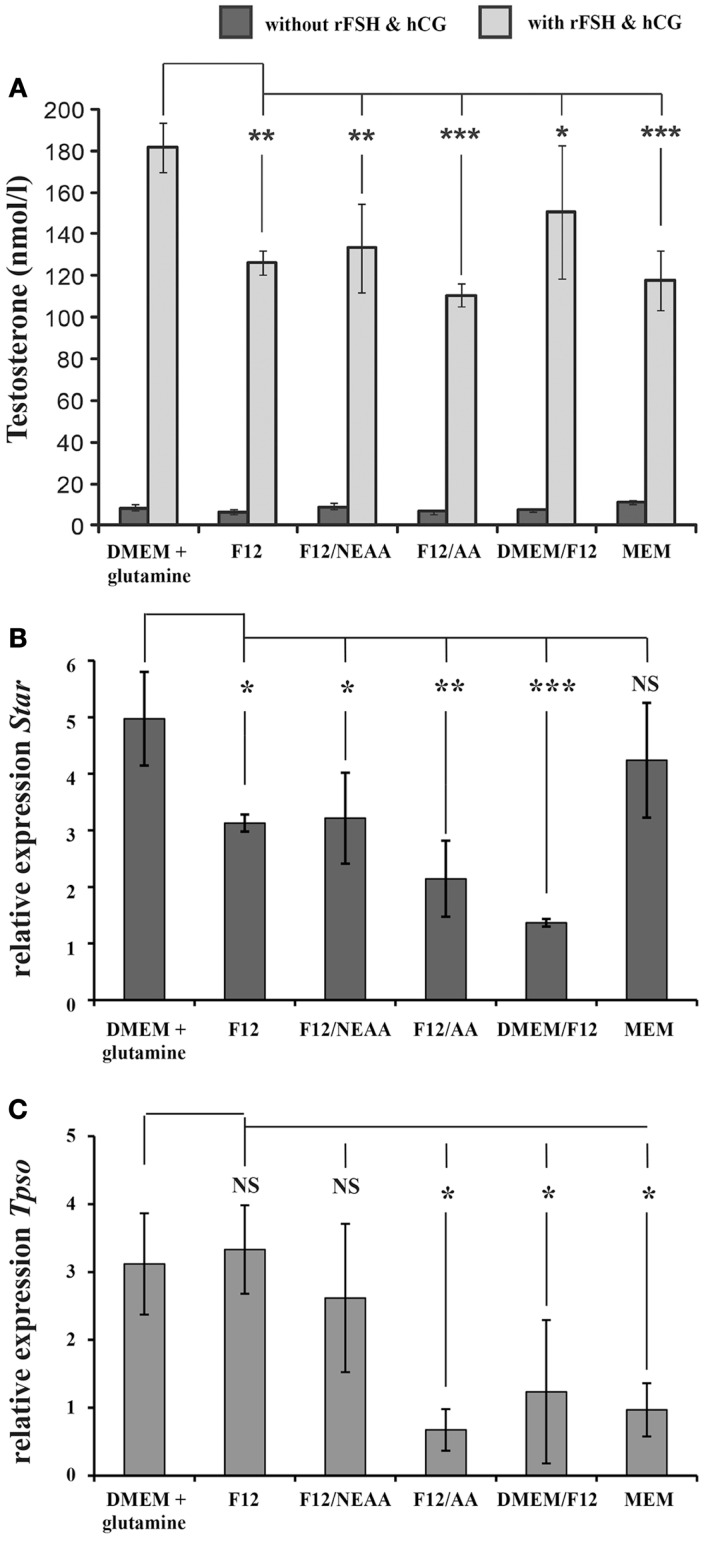
**The influence of various culture media on the capacity of the Leydig cells in three-dimensional cultures of rat testicular cells to produce testosterone and express steroidogenic genes**. **(A)** On the *X*-axis are the different culture media employed [DMEM + glutamine (GL), F12, F12 + NEAA (non-essential amino acids), F12 + AA (essential amino acids), F12/DMEM, and MEM (minimal essential medium)], and the *Y*-axis depicts the concentration of testosterone (evaluated by radioimmunoassay and expressed in nanomoles/liter) in the medium of cells cultured for 1 day. The relative expression of **(B)** *Star* (Steroidogenic Acute Regulatory Protein) and **(C)** *Tspo* (Translocator Protein) (determined by qPCR analysis with *Actb* as an internal control) by testicular cell suspensions from 7 d*pp* rats cultured for 1 day in six different media in the presence (light columns) or absence (dark columns) of hCG and FSH. The mean relative expression for triplicates was calculated by the ddCt procedure. One-way ANOVA with the Shapio–Wilk test for normality was applied to compare the different experimental conditions. NS: non-significant; *p < 0.05, **p < 0.01, ***p < 0.001 in comparison to the value with DMEM + glutamine.

Comparison of the production of testosterone during the first 24 h of *in vitro* culture with stimulation by gonadotropins revealed significantly lower testosterone levels with F12, DMEM/F12, and MEM media than with DMEM + glutamine (Figure 1). According to the supplier, DMEM + glutamine contains higher levels of amino acids than F12 and DMEM/F12, but addition of NEAA or AA to the F12 medium did not elevate testosterone production to the same level as with DMEM + glutamine (Figure 1A). For all media examined, testosterone production was stimulated by gonadotropins, as expected (Figure 1A).

### Influence of the various culture media on the expression of steroidogenic genes by Leydig cells in three-dimensional cultures

As assessed by qPCR, within 1 day of stimulation by gonadotropins the relative up-regulation of *Star* expression was fivefold with DMEM + glutamine, threefold with F12, threefold with F12/NEAA, twofold with F12/AA, onefold with DMEM/F12, and fourfold with MEM (Figure 1B). The increase with DMEM + glutamine was significantly higher than with all of the other culture media except MEM. Moreover, after 1 day of stimulation with gonadotropins, the relative expression of *Tspo* was also up-regulated (DMEM + glutamine, threefold; F12, threefold; F12/NEAA, threefold; F12/AA, onefold; DMEM/F12, onefold; and MEM, onefold) (Figure 1C). This elevation was significantly greater with DMEM + glutamine than F12/AA, DMEM/F12 or MEM. Thus, with DMEM + glutamine, up-regulation of both *Star* and *Tspo* was most pronounced, in agreement with the observation that testosterone production was highest in the same medium.

### Influence of the various culture media on testosterone production by Leydig cells in three-dimensional cultures

**Figure 2:**
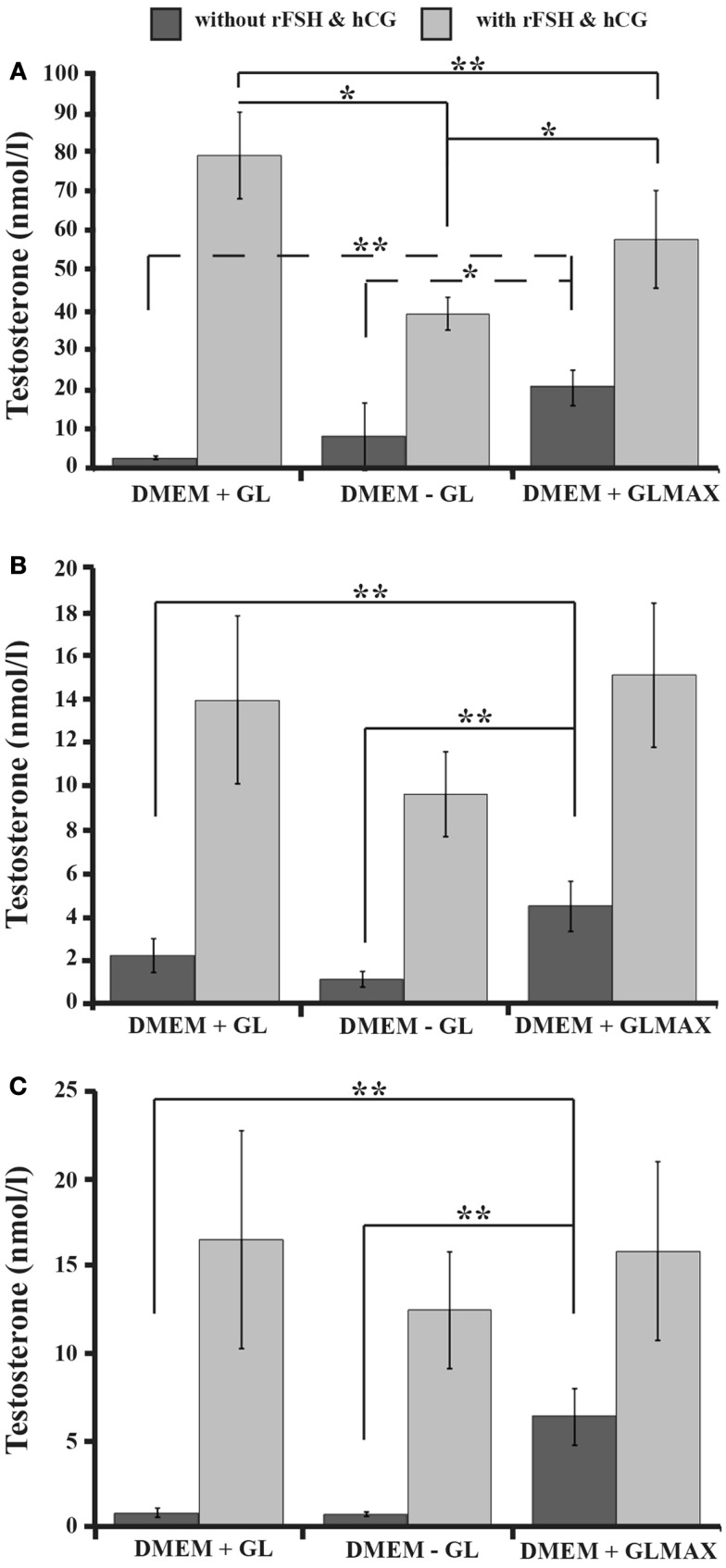
**The influence of different DMEM culture media on testosterone production by the Leydig cells in three-dimensional cultures of rat testicular cell**. The cells were cultured for 14 days in DMEM + glutamine (GL), DMEM − glutamine (GL), or DMEM + Glutamax (GLMAX) (presented on the *X*-axis) either with (light columns) or without (dark columns) hCG and rFSH stimulation. The concentration of testosterone in the culture medium following 1 day **(A)**, 7 days **(B)**, and 14 days **(C)** of culture (determined by radioimmunoassay and expressed in nanomoles/liter) is shown on the *Y*-axis. One-way RM ANOVA with the Shapio–Wilk test for normality was applied to compare the different experimental conditions. *p < 0.05,**p < 0.01.

The functionality of the Leydig cells in the mixture of testicular cells was assessed on the basis of testosterone production after 1, 7, and 14 days of culture, both in the presence and absence of gonadotropins. There was a significant difference between stimulated and un-stimulated cells at all three time-points with DMEM + glutamine, DMEM without glutamine (−glutamine), or DMEM + Glutamax. After 1 day of stimulation this production was highest with DMEM + glutamine, followed by DMEM + Glutamax, and the lowest level with DMEM − glutamine (Figure 2A), but there was no significant difference between these three media in this respect following stimulation for 7 or 14 days (Figures 2B,C). At the same time, DMEM + Glutamax promoted the capacity of basal (unstimulated) Leydig cells to produce testosterone after 1, 7, and 14 days to a greater extent than DMEM+ or −glutamine (Figures 2A–C). Moreover, the levels of testosterone after 1 day of culture in all three media with gonadotropins (DMEM + glutamine: 79 ± 11 nmol/l; DMEM − glutamine: 39 ± 4 nmol/l; DMEM + Glutamax: 58 ± 12 nmol/l) as well as in DMEM + Glutamax without stimulation (20 ± 6 nmol/l), were higher than after 7 days (DMEM + glutamine: 14 ± 4 nmol/l; DMEM − glutamine: 10 ± 2 nmol/l; DMEM + Glutamax: 15 ± 3 nmol/l; DMEM + Glutamax without stimulation: 4 ± 1 nmol/l) or 14 days (DMEM + glutamine: 16 ± 6 nmol/l; DMEM − glutamine: 12 ± 3 nmol/l; DMEM + Glutamax: 16 ± 5 nmol/l; DMEM + Glutamax without stimulation: 6 ± 2 nmol/l) of culture (Figures 2A–C).

### Gonadotropins protect rat testicular cells in the different culture media from apoptosis

**Figure 3:**
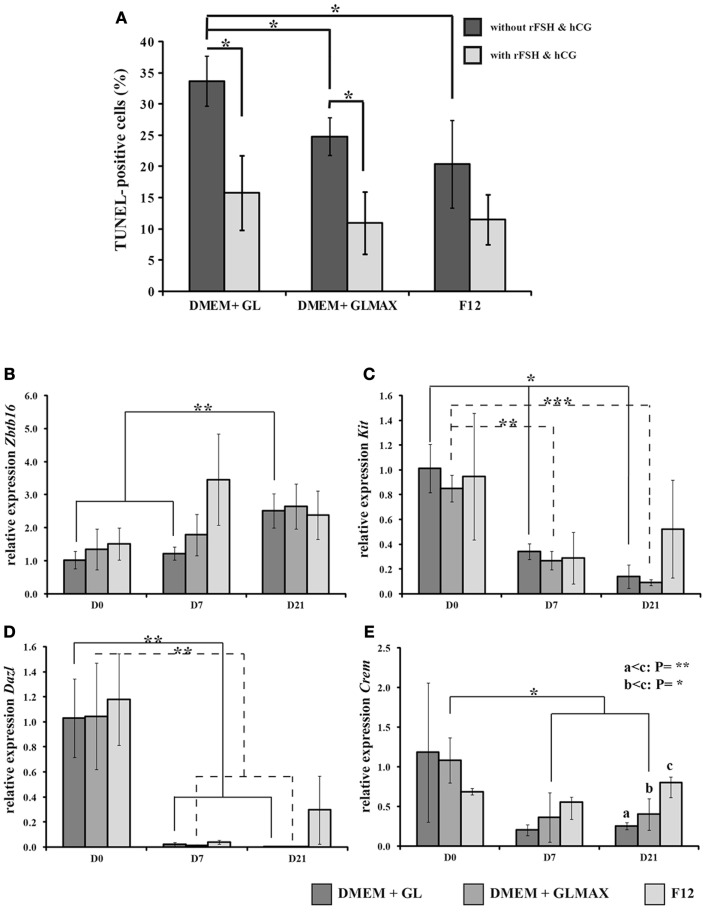
**The influence of different culture media on cell survival and expression of genes related to germ cell differentiation in a three-dimensional cultures of rat testicular cells**. **(A)** The cells were cultured for 7 days in DMEM + glutamine (GL), DMEM + Glutamax (GLMAX), or F12 (presented on the *X*-axis) either with (light columns) or without (dark columns) hCG and rFSH stimulation. The percentage of apoptotic (TUNEL-positive) cells, normalized to the 1-day value, is shown on the *Y*-axis. **(B–E)** The cells were cultured for 0, 7, and 21 days. The graphs depict the relative expression of rat *Zbtb16* (also known as *Plzf*) **(B)**, *Kit* **(C)**, *Dazl* **(D)**, and *Crem* **(E)** (determined by qPCR analysis with *Actb* as an internal control) by cells cultured in DMEM + glutamine (DMEM + GL), DMEM + Glutamax (DMEM + GLMAX), or F12. On the *X*-axis, the different periods of culture [0 (D0), 7 (D7), and 21 (D21) days] are depicted and the *Y*-axis shows the mean relative expression of replicates calculated by the ddCt procedure. Student’s *t*-test was applied to compare the different experimental conditions. *p < 0.05, **p < 0.01, ***p < 0.001.

Cell proliferation, expressed as the percentage of Ki67 positive cells (%) after 1 day of culture was 3.2 ± 0.8 with DMEM + glutamine, 3.9 ± 0.9 with DMEM + Glutamax, and 2.6 ± 2.6 with F12, with no significant differences. Nor did the relative numbers of different cell types immediately following the enzymatic digestion and after 1 day of culture differ between the culture media examined (DMEM + glutamine: 82 ± 17% Ddx4-positive cells, 32 ± 10% Vimentin-positive cells, 2 ± 1% 3 βHSD-positive cells; DMEM + Glutamax: 77 ± 13% Ddx4-positive cells, 28 ± 10% Vimentin-positive cells, 3 ± 2% 3 βHSD-positive cells; F12: 92 ± 6% Ddx4-positive cells, 26 ± 9% Vimentin-positive cells, 1 ± 1% 3 βHSD-positive cells). Application of the TUNEL assay revealed a significantly lower rate of apoptosis following 7 days of culture with than without gonadotropins in DMEM + glutamine (15 vs. 33%) or DMEM + Glutamax (10 vs. 24%) (Figure 3A), but no such difference was observed in the case of the F12 medium. Without stimulation, the cells in DMEM + glutamine exhibited a higher apoptotic rate (33%) than those in DMEM + Glutamax (24%) or F12 (20%), whereas there was no such difference when these three media were supplemented with gonadotropins (Figure 3A).

### Medium-related effects on the differentiation of pre-pubertal rat male germ cells *in vitro*

When expression of *Zbtb16* (also known as *Plzf*), *Kit*, *Dazl*, *Boll*, *Crem*, and *Protamine* by cells cultured with hCG and FSH was evaluated by qPCR, the expression of *Zbtb16* in DMEM + glutamine was observed to be significantly higher (2.5-fold) after 21 days than after 0 and 7 days, with no such changes in the case of DMEM + Glutamax or F12 and no significant differences between these three different media (Figure 3B). With DMEM + glutamine or DMEM + Glutamax, *Kit* expression was down-regulated after 7 (threefold) and 21 days (fivefold) in culture, whereas in cells cultured in F12 this expression remained constant during the entire experimental period (Figure 3C). Expression of *Dazl* by cells cultured in DMEM + glutamine or DMEM + Glutamax was significantly down-regulated (10-fold) after 7 and 21 days with a similar, although not significant tendency in the case of F12 (11- and 3-fold down-regulation after 7 and 21 days, respectively) (Figure 3D). After 7 days, only cells in DMEM + Glutamax demonstrated down-regulation (2.5-fold) of *Crem* expression (Figure 3E), while after 21 days, expression of *Crem* was significantly higher with F12 than DMEM + glutamine (threefold) or DMEM + Glutamax (twofold) (Figure 3E). No expression of *Boll* or *Protamine* was detected under any of the experimental conditions (data not shown).

### Morphological evaluation and immunohistochemical and fluorescent staining

**Figure 4:**
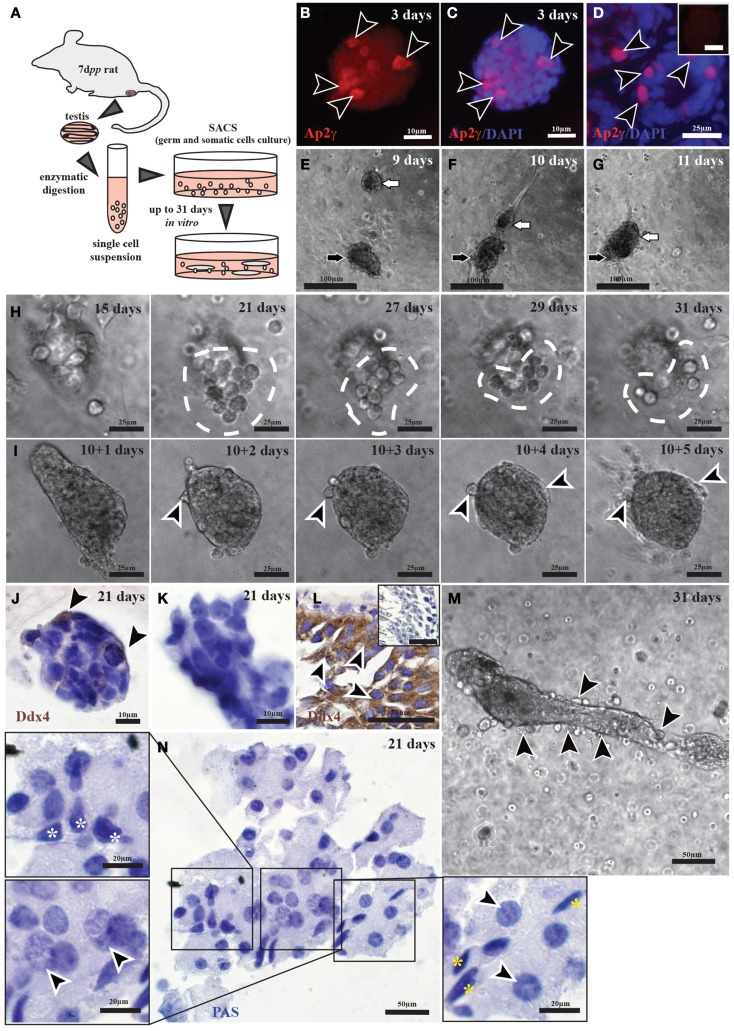
**Tubule formation by and germ cell differentiation of pre-pubertal rat testicular cells in three-dimensional cultures**. **(A)** A schematic overview of the experimental conditions. **(B–D)** Immunofluorescent staining of undifferentiated spermatogonia (Ap-2γ) in cell colonies originated from culturing cells from 7-day-old rats for 3 days **(B,C)**, as well as from 8-day-old rats as a positive control **(D)** [Ap2γ: red staining (black arrow heads); DAPI: blue staining]. The negative control with IgGs is shown as small insert in **(D)**. **(E–G)** 9- **(E)**, 10- **(F),** and 11-day cultures **(G)** showing two colonies (the black and the white arrows) migrating toward one another. **(H,I,M)** Active migration of cells out of colonies cultured for as long as 31 days [white dashed line in **(H)**, black arrows heads in **(M)**] or following incubation of isolated cell colonies in liquid medium for 5 days [black arrow heads in **(I)**]. **(J–L)** Immunohistochemical staining for germ cells (Ddx4) in colonies originating from 7-day-old rats and cultured for 21 days **(J,K)**, as well as from 60-day-old rat testis [positive control; **(L)**] [Ddx4: brown staining (black arrow heads); Hematoxylin: blue staining]. **(N)** Cells cultured for 21 days exhibit morphologies similar to those of peritubular cells (yellow stars), Sertoli cells (white stars), leptotene spermatocytes, and early pachytene spermatocytes (black arrow heads).

Morphological evaluation and IHC and IF staining revealed colony formation in the three-dimensional cultures of rat testicular cells (Figure 4A), with undifferentiated spermatogonia being detected in these colonies (Figures 4B–D). Following 3 days in culture, the colonies formed by un-stimulated cells were already less compact than those formed in the presence of gonadotropins (data not shown). Active cell migration toward colonies could be observed (Figures 4E–G). However, the total number of viable cell colonies was low.

Morphological evaluation of colonies formed in the three-dimensional culture (Figures 4H,M), as well as in conventional two-dimensional cultures (Figure 4I) revealed migration of cells from the inner side to the outer side of the colonies. These migrating cells could be identified as germ cells by IHC staining for Ddx4, a marker specific for germ cells (Figures 4J–L).

More detailed morphological analysis after 21 days *in vitro* showed small structures containing a mixture of Sertoli (Figure 4N) and peritubular cells (Figure 4N), as well as male germ cells in different stages of differentiation up to early pachytene spermatocytes (Figure 4N).

## Discussion

The major novel observations documented here are as follows: (1) the culture medium *per se* exerts a direct influence on the functionality of the rat Leydig cells, but not on germ cell differentiation in three-dimensional cultures; (2) rat germ cells migrating from the inner side to the outer side of the cell colonies suggest an unfavorable organization of tubules formed *de novo* in the three-dimensional culture; (3) undifferentiated rat spermatogonia differentiate up to the stage of pachytene spermatocytes in a similar time-period to the situation *in vivo* in three-dimensional cultures.

After 7 days of culture in three different media, less extensive apoptosis was observed among cells in the presence than in the absence of rFSH and hCG, in agreement with earlier findings in literature (18–20). The nature of the medium *per se* exerted no significant impact on overall cell survival.

Although in our three-dimensional cultures stimulation with gonadotropins promoted Leydig cell functionality (as reflected in testosterone production) after 1, 7, and 14 days regardless of the medium, DMEM + glutamine was clearly most effective in this respect after 1 day of stimulation. Thus, at this early time-point, the level of testosterone in the culture medium appeared to be related to the levels of glutamine [an important source of energy, as well as a precursor for protein synthesis (21–24)], since the other culture media examined contain less glutamine or none at all. In addition, cells cultured in DMEM + glutamine exhibited the most pronounced up-regulation of *Star* and *Tspo*, which transfer cholesterol (the precursor for testosterone) across an aqueous phase from the outer to the inner mitochondrial membrane (25–28) and are thereby essential for the steroidogenic process. Thus, the presence of glutamine in the culture medium may be essential for the synthesis of the enzymes and other proteins required for testosterone production.

Furthermore, since DMEM + glutamine medium contains higher levels of amino acids both (essential and non-essential) than F12, this difference was eliminated by adding essential or non-essential amino acids to the F12 medium. However, such supplementation did not increase testosterone production to a level similar to that obtained with DMEM + glutamine and addition of both kinds of amino acids to F12 resulted in a low pH and thereby a cytotoxic environment (data not shown). Moreover compared to DMEM + glutamine, the relative levels of expression of *Star* and *Tspo* were lower in cells cultured in F12 supplemented with essential amino acids, and expression of *Star* was lower when F12 was supplemented with non-essential amino acids medium. Of course, DMEM + glutamine and F12 also differ with respect to the levels of several other components, such as vitamins and inorganic salts, which might explain their different effects.

Analysis of the relative expression of genes associated with male germ cell differentiation (i.e., *Zbtb16*, *Kit,* and *Dazl* in spermatogonia, *Dazl* and *Boll* in spermatocytes, and *Crem* and *Protamine* in spermatids) by cells cultured in DMEM + glutamine, DMEM + Glutamax, or F12 supplemented with gonadotropins demonstrated that none of these media alone promoted robust spermatogenesis after 21 days of culture. The overall down-regulation of these genes might reflect the increase in the number of apoptotic cells with culture time, which could also explain at least partially the low efficiency of the three-dimensional culture system employed.

However, there were certain differences in the expression of *Crem* by cells in the different media after 21 days. *Crem* is expressed primarily by spermatocytes, but also by Sertoli cells, although the latter expression appears not be necessary for spermatogenesis (29–32). *Crem* acts downstream of cAMP signaling (33) and its activation modulates the cAMP response element, thereby altering gene expression (32, 33). Interestingly, the different isoforms of the Crem protein act as a master switch for the regulation of various genes during spermatogenesis (30–32, 34).

After 21 days in culture, the cells in only a few of the colonies formed still exhibited an intact morphology, most having decreased in size. However, all colonies with intact cells contained a mixture of somatic (Sertoli and peritubular cells) and germ cells (differentiated as far as pachytene spermatocytes). Thus, undifferentiated spermatogonia, the only germ cells present in the testes of 7 d*pp* rats had differentiated as far as to the stage of pachytene spermatocytes, a level of differentiation similar to the situation *in vivo* at the age of 25–28 d*pp*. These observations indicate that at least a partially functional microenvironment supporting germ cell survival and differentiation was obtained.

Suitable support for germ cells through the formation and proper orientation of Sertoli and peritubular cells is needed for completion of spermatogenesis (13, 35, 36). As shown earlier, when utilized as feeders for germ cells or embryonic stem cells *in vitro*, Sertoli cells tend to be unorganized in contrast to their highly polarized orientation *in vivo* (35–37). Such disorganization presumably disallows the crucial support of the blood–testis barrier as a result of missing or premature junctional complexes between Sertoli cells (36). Such lack of support leads to meiotic arrest, with the meiotic germ cells going into apoptosis.

In our three-dimensional cultures, germ cells were seen to migrate out of the cell colonies formed and thereafter disintegrate and die within a couple of days due to the lack of support from the Sertoli cells. Strategies for obtaining the proper polarized orientation of the Sertoli cells and thereby establishing an appropriate niche for germ cell differentiation *in vitro* warrant more detailed investigations.

In conclusion, the present study demonstrates that although the nature of the culture medium *per se* does not influence the overall viability of rat testicular cells *in vitro*, it does influence the functionality of rat Leydig cells in three-dimensional cultures. Cells cultured in DMEM + glutamine medium displayed more testosterone production and higher expression of *Star* and *Tspo* than any of the other cell culture media examined. This might reflect the higher concentration of glutamine in this medium, but further studies concerning the influence of glutamine on Leydig cell functions, as well as on other endocrine/paracrine pathways in such complex three-dimensional cultures containing all types of testicular cells are required.

Differentiation of germ cell up to the stage of pachytene spermatocytes, i.e., similar to the situation *in vivo*, could be detected in a few small colonies hosting a mixture of somatic and germ cells. However, the crucial structural support provided by the Sertoli and peritubular cells in the seminiferous tubules *in vivo* could not be duplicated and none of the media examined provided a robust system for male germ cell differentiation *in vitro*. Thus, additional work on this question remains to be done.

## Author Contributions

Ahmed Reda: study design, data acquisition, analysis and interpretation, drafting the article, and final approval of the submitted version. Mi Hou, Luise Landreh, Kristín Rós Kjartansdóttir: data acquisition and analysis, drafting the article, and final approval of the submitted version. Konstantin Svechnikov, Olle Söder: data analysis and interpretation, drafting the article, and final approval of the submitted version. Jan-Bernd Stukenborg: study design, data acquisition, analysis and interpretation, drafting the article, and final approval of the submitted version.

## Conflict of Interest Statement

The authors declare that the research was conducted in the absence of any commercial or financial relationships that could be construed as a potential conflict of interest.
